# Three-Dimensional Interaction Homology: Deconstructing Residue–Residue and Residue–Lipid Interactions in Membrane Proteins

**DOI:** 10.3390/molecules29122838

**Published:** 2024-06-14

**Authors:** Glen E. Kellogg

**Affiliations:** Department of Medicinal Chemistry, Virginia Commonwealth University, Richmond, VA 23298-0540, USA; glen.kellogg@vcu.edu

**Keywords:** hydropathic interactions, solvent-accessible surface area, membrane proteins, lipid interactions

## Abstract

A method is described to deconstruct the network of hydropathic interactions within and between a protein’s sidechain and its environment into residue-based three-dimensional maps. These maps encode favorable and unfavorable hydrophobic and polar interactions, in terms of spatial positions for optimal interactions, relative interaction strength, as well as character. In addition, these maps are backbone angle-dependent. After map calculation and clustering, a finite number of unique residue sidechain interaction maps exist for each backbone conformation, with the number related to the residue’s size and interaction complexity. Structures for soluble proteins (~749,000 residues) and membrane proteins (~387,000 residues) were analyzed, with the latter group being subdivided into three subsets related to the residue’s position in the membrane protein: soluble domain, core-facing transmembrane domain, and lipid-facing transmembrane domain. This work suggests that maps representing residue types and their backbone conformation can be reassembled to optimize the medium-to-high resolution details of a protein structure. In particular, the information encoded in maps constructed from the lipid-facing transmembrane residues appears to paint a clear picture of the protein–lipid interactions that are difficult to obtain experimentally.

## 1. Introduction

Understanding and exploiting protein structure has been a major goal of a dedicated and large group of biologists for nearly three-quarters of a century. In 1951, Linus Pauling suggested both the α-helix and β-strand motifs [1] almost a decade before they were experimentally observed by John Kendrew [2,3] and Max Perutz [4,5] in the crystal structures of myoglobin and hemoglobin. These *secondary* structure motifs are certainly crucial to protein structure, but many other features, both short and long ranges, significantly contribute to the diversity of observed protein structures. In other words, there is far more to protein structure than hydrogen bonding. In particular, the close examination of residue–residue interactions, such as that by Juswinder Singh and Janet Thornton [6], reveals a compelling collection of interaction types. The interactions designated as being hydrophobic are perhaps most interesting: while, at first, they are “obvious”, this characterization belies their complexity as they are actually emergent properties involving enthalpy, entropy, and solvation [7]. It should be noted that Irving Klotz [8] highlighted the importance of hydrophobic phenomena in proteins before the first crystal structures were reported.

Nearly all of the solved crystal structures for the first fifty or so years of protein structural biology were for soluble proteins as they can be (relatively) easily crystallized with conventional methods. Membrane protein structural biology has a number of additional challenges, not least of which is that their structure (and function) is likely to be highly affected by the integrity of the lipid bilayer in which they are embedded. Underscoring this importance, Qin et al. [9] showed nearly two decades ago that the lipids surrounding a membrane-bound binding site have conserved “binding sites”. A second revolution in structural biology was ignited as various therapeutically important G protein-coupled receptor (GPCR) and ion channel structures were solved by a number of brilliant strategies to circumvent Nature. Notably, many of these techniques involved the detergent-based extraction of the protein from its native membrane and reinsertion into a substitute simulated bilayer [10,11,12,13]. The current excitement for Cryo-EM structural biology is based in large part on the promise of more native-like membrane structures. However, even Cryo-EM has similar issues as protein extraction is still a fraught process. Thus, native or even reasonably similar lipids performing the same structural roles are rarely present in crystals or cryo-EM particles, and misinterpretations of reported structures have been published [14,15,16,17]. The emerging ability to examine single particles, potentially encapsulated in a native-like “bundle” of lipids, is exciting [18]. A recent review by Levental and Lyman [19] describes the current state of understanding of protein–lipid interactions, while another two reviews from 2019 by Corradi et al. [20] and 2020 by Duncan, Song, and Sansom [21] focus on what has been learned from experimental and simulated structures. 

A further exciting development is the maturation of computational de novo protein structure prediction tools, such as AlphaFold [22,23], Rosetta [24,25], I-TASSER [26], and ESMFold [27]. These tools incorporate substantive information from protein sequence homology wrapped around sophisticated interaction scoring algorithms. Very impressive predictive structure models have been reported [28], although smaller-scale predictions, such as rotamer conformation, etc., are not yet handled well enough to reliably apply such predicted structures for drug discovery. Also significant is that these programs have been developed mostly based on soluble proteins, and when membrane proteins were included in the training, those structures did not contain lipid bilayers, etc., and can conceivably even be flawed due to the aforementioned issues with detergent extraction [14]. Very recent computational methods have hybridized AlphaFold2 and other deep learning/modeling methods, such as Rosetta, I-TASSER, etc., with Cryo-EM density maps to build better protein structure models, e.g., DeepMainmast [29], DEMO-EM2 [30], and DeepTracer [31]. None of these methods, however, have any facility for modeling membrane lipids.

Our contribution to modeling and understanding protein structure, which we have been developing over the past decade, has as its hypothesis that it is the residue character, especially their three-dimensional interaction networks, that is the determinant of protein structure. While self-evident, our hypothesis de-emphasizes sequence homology in favor of a residue-level *hydropathic valence*, which is the set of interactions each residue ideally would make: involving interaction type (both favorable—like hydrophobic and hydrogen bonds—and unfavorable—like repulsive Coulombic and desolvation), interaction strength, and their 3D spatial arrangement. We encoded these interaction sets in 3D contourable hydropathic interaction maps calculated using a feature of the HINT (Hydropathic INTeractions) modeling system [32,33]. Crucially, after clustering, there are a limited number of unique maps and they are dependent on the residue type and backbone angles. Our development of this paradigm was evolutionary, and we reported our progress with a series of articles targeting single residue types or families, starting with tyrosine [34], followed by alanine [35]; phenylalanine, tyrosine, and tryptophan [36]; aspartic acid, glutamic acid, and histidine [37]; serine and cysteine [38]; and alanine, isoleucine, leucine, proline, and valine [39]. We extended the scope of this paradigm in the latter pair of studies by profiling a second dataset of membrane proteins that were exhaustively optimized, studied, and made available in the MemProtMD database [40] of intrinsic membrane protein structures from the Protein Data Bank, followed by molecular dynamics optimization within simulated lipid bilayers (dipalmytoylphospha-tidylcholine, DPPC). We applied several filters from the MemProtMD database to populate three residue subsets: first, residues, especially in intracellular and extracellular loops, helices, etc., do not interact with the bilayer and are quite similar to their analogues in soluble proteins; second, residues at the core of the trans-membrane region, in channels or GPCR binding sites, also have minimal direct interactions with lipids; and third, a set constituting the residues that do interact with the lipids molecules in the bilayer. We termed these three residue subsets (or environments) as mS (soluble), mC (core), and mL (lipid-facing), respectively. 

In the 2023 report [39], we only considered the aliphatic hydrophobic residues and characterized their interactions in soluble proteins and in the three structural environments described above. We also characterized the roles of these residues by applying the GETAREA [41] algorithm to obtain the sidechain solvent-accessible surface area (SASA), supplemented with a relatively simple adaptation to calculate a new parameter: the lipid-accessible surface area (LASA) for the mL residue subset [39]. Lastly, we calculated the hydropathic interaction maps for the mL subset in two different ways: (1) including the interactions with the lipid molecules (mL), which was the basis for map clustering, and (2) by turning off those interactions (mN). The difference between the maps created using those two protocols would, then, represent only residue–lipid interactions. We hypothesized that such information would be invaluable in building molecular models for membrane proteins that include bilayer molecules, perhaps to the extent that they could supplement current protein structure prediction methods in membrane protein cases.

In this paper, we report that the membrane protein calculations are complete for all residue types. We describe the scope of the data, including metrics illuminating the SASA, LASA, and hydropathic interaction character and preferences of the transmembrane residues by type. For this work, we also completed residue characterization by calculating a metric that quantitates the interaction character for each residue map cluster (and residue type) in the various datasets. We illustrate the information content of these maps, which are a deconstruction of sets of interactions and forces leading to protein structure in a quantitative, visual, and intuitive manner for both soluble and membrane-bound proteins. The volume of data involved in this work allows us only to present sample results, but significantly more information is available in Supporting Information.

## 2. Results and Discussion

### 2.1. Characteristics of the Datasets

Our goal was to explore a variety of environments within proteins that represent the structural motifs found in soluble and membrane-bound proteins. In earlier work, we suggested that dividing the 2π × 2π Ramachandran plot for protein backbone angles into sixty-four π/4 × π/4 “chess squares” was an appropriate methodology for determining and cataloguing backbone dependence for sidechain conformational differences, but more importantly sidechain interaction preferences [34]. We later implemented [36,37] a chess square parsing scheme for larger residues to filter sidechain conformations into 60 ± 30°, 180 ± 30°, and 300 ± 30° χ_1_ bins for ASN, ASP, CYS, HIS, ILE, LEU, MET, PHE, SER, THR, TRP, and TYR, and these were further and similarly subdivided into (9) χ_1_/χ_2_ bins for ARG, GLN, GLU, and LYS. PRO residues were parsed into two bins centered around χ_1_ = +30° and –30°, while ALA, GLY, and VAL were not parsed. Our nomenclature is that chess squares are named ***a1***–***h8***, from lower left to upper right on the Ramachandran plot, and are always written in bold italic font. The parses are named (for all residues except PRO) ***.60***, ***.180***, and ***.300***, and are appended to the chess square to identify a bin (for ARG, GLN, GLU, and LYS, these names are ***.60.60***, ***.60.180***, etc.). PRO parses are named as ***.30m*** and ***.30p*** [36]. The -S-S- bridged cysteine (CYX) was treated as described earlier [38].

Later, as mentioned above, we added a second collection of proteins to our studies that focused on those membrane-bound by abstracting a portion of the MemProtMD database [40] (http://memprotmd.bioch.ox.ac.uk (accessed on 22 May 2023)) and used the filters thus provided to build three subsets of residues: (1) extracellular, i.e., in the soluble domain, that we termed the mS dataset; (2) intracellular and lipid-facing, termed the mL dataset; and (3) intracellular and core-facing, termed the mC dataset. An alternative interaction calculation method, wherein interactions between the residues and lipids are ignored, was termed the mN dataset.

Table 1 summarizes the data in terms of the numbers of residues in each dataset. The soluble dataset is the largest, encompassing nearly 750,000 residues with occurrence frequencies generally in line with the accepted norm. Altogether, there are about half as many residues in the membrane-bound protein datasets, but these are split unevenly: the mS dataset contains 54% of the residues, the mC dataset contains only 7% of the residues and the mL dataset contains the remaining 39%. In agreement with the expectation that the mS dataset comprises residues much like those found in soluble proteins, the mS occurrence frequencies are in reasonable agreement with those found in the soluble dataset. However, the mC dataset shows some significant and interesting differences (compared to mS): (1) It is much less likely to find an ARG, ASP, GLU, LYS, or PRO as a core-facing residue. This is interesting because these, other than PRO, are most of the residues associated with some sort of regulatory function (PRO is probably just ill-suited for the structural constraints of the core); (2) The most prominent increase in occurrence in the mC dataset is exhibited by PHE, and to a lesser extent by TYR, suggesting a necessary π-stacking interaction role here; and (3) There are no CYX residues and only a small number of CYS residues. Even more dramatic, but largely expected, are the differences between relative populations in the mL dataset compared to mS. The hydrophobic residues, ALA, ILE, LEU, PRO, and VAL, along with PHE, are now 57% of the residues found as lipid-facing (vs. 40% in the mS dataset). In the mL dataset, the four formally-charged residues ARG, ASP, GLU, and LYS now comprise just 7% (vs. 25% in the mS dataset). 

### 2.2. Three-Dimensional Interaction Maps and Map Clustering

To catalogue the array of interactions for each residue’s sidechain—that shape its conformation and role in protein structure at all scales—we calculated three-dimensional maps that enumerated the interactions between that residue and its environment, i.e., the rest of the protein and cofactors, such as water. Hydrophobic, hydrophobic-polar, favorable polar such as hydrogen bonding and acid–base, and unfavorable polar such as acid–acid and base–base interactions were scored such that their strengths are mapped in space [34,35]. These maps were binned by the residue type, chess square, and, as appropriate (vide supra), χ_1_ and χ_2_ parses so that their sidechains were aligned [37]. 

Next, our pairwise similarity metric [34] was applied to each map pair in its bin, and these metrics were set into a two-dimensional matrix in preparation for clustering. We used the k-means unified gap method [42] based on the testing of a numerous other methods [34], and manually set the maximum number of clusters based on our early experiences to maximize the likelihood that the residue types and/or chess squares we wished to compare would have corresponding cluster counts [39]. Table 1 indicates the number of clusters found for each residue type in each dataset. These vary over a wide range and are dependent on the number of residues present (weakly populated datasets will have fewer), the number of parses (ALA has one, while LYS has nine), and the structural complexity that in turn dictates the maximum allowed (ALA-4, ARG-18, ASN-12, ASP-12, CYS-6, CYX-12, GLN-12, GLU-12, GLY-9, HIS-12, ILE-9, LEU-9, LYS-18, MET-18, PHE-12, PRO-6, SER-6, THR-6, TRP-12, TYR-12, and VAL-9). It was our hypothesis that these cluster-derived and weighted-average map sets represent a significant reduction in data compared to the original structural data (at least 50-fold in the soluble dataset), and that we have sampled enough that it is unlikely that many new motifs would appear with larger protein datasets. For classification, each cluster is identified by the most representative residue member (in an ordinal list), which is called the exemplar, and is the residue (map) closest to the cluster’s centroid. Our nomenclature is that cluster names are written in bold font. Thus, in our approach, one member within the set of average maps in the specified backbone conformation very likely captures the constellation of interactions between any residue and its environment. In other words, this is the “hydropathic valence” of the residue type in its secondary structure (chess square). To summarize, these maps are conserved motifs that are information-rich backbone-dependent rotamer and interaction libraries. 

Our earlier articles [34,35,36,37,38,39] provide examples of cluster-derived hydropathic interaction maps like those described above. We will show a handful of targeted cases below.

### 2.3. Solvent-Accessible and Lipid-Accessible Surface Areas 

To understand further the role each residue plays in structure, we calculated the solvent-accessible surface area (SASAs) for all residue sidechains using the GETAREA algorithm and server [41]. The SASA is a metric representing the degree of exposure/buriedness of a residue’s sidechain and at its basic level reveals the residue’s position within the protein, i.e., surface or not. It becomes a more powerful structure-understanding tool when the identity of the residue is considered: high solvent exposure in a hydrophobic residue has an entirely different meaning than high solvent exposure in a polar residue. This is particularly relevant in considering membrane-bound proteins because lipid-facing residues are generally hydrophobic rather than polar but have large calculated SASAs because the membrane bilayer is not per se protein and thus not recognized by GETAREA. For this reason, we implemented an adjustment, which we call lipid-accessible surface-area (LASA), where accessible surface area is categorized as SASA or LASA based on the ratios of score sums involving atoms in the DPPC molecules to the total score.

Table 2 sets out the average solvent-accessible and lipid-accessible surface areas by residue type for the sidechains of the four datasets. Also listed are the reference random coil SASAs for Gly-X-Gly tripeptides [41]. Notes: (1) As GLY does not have a sidechain, its actual SASA is 0.0. We did all of our calculations on its full structure and the random coil value listed is that value; (2) Bridging cysteine (CYX) is not recognized by GETAREA, and our calculations actually consider each half of CYX to be –CB-SG-SG’-CB’. The random coil number presented in Table 2 is arbitrarily 150% of the CYS random coil SASA. These two values will only be used as normalization factors in the calculations below.

While most indications are that the structural characteristics and the residues themselves are very similar between the soluble dataset and the mS dataset, there are notable differences in the average SASAs between the two. While we cannot be definitive, it seems likely that this is due to the different ways the structure models within the two datasets were collected and processed. In general, for smaller residues (ALA, CYS, ILE, LEU, SER, and VAL), the SASAs are largely the same, but for most of the larger residues (ARG, GLN, GLU, HIS LYS, TRP, and TYR), the mS dataset residues are less solvent-exposed. Since the latter set was subjected to extensive molecular dynamics, it may be that the larger sidechains were optimized into more compact conformations that may be less native-like. 

For the mC and mL datasets, we calculated both residue-average SASAs and LASAs. The mC LASA data account for 4% to 17% (average 9%) of the total accessible surface areas for these residues. This must represent residues whose backbones are in the core region but whose sidechains make contact with lipid molecules. The three residue types with the largest LASAs are somewhat unexpected: HIS, MET, and PHE. Perhaps, the sidechains of aromatic planar residues (TRP but not TYR also has a high LASA) have affinity and access to the lipid. Interestingly, only the MET of the long chain residues reaches the lipids: ARG, GLN, GLU, and LYS have below-average LASAs, likely because they are less hydrophobic.

In the mL dataset, the LASA captures 72% of the accessible surface area, with largely expected trends. The larger hydrophobic residues (including PHE and TRP) show between 84% and 89% LASA, while in contrast, ASN, ASP, GLN, and GLU show between 49% and 60% LASA. ALA and PRO likely have less prominent LASAs, 76% and 77%, respectively, because they are making a larger fraction of their interactions with neighboring residues. In the following section, we will examine the interaction character for each residue type and dataset.

Examining the SASA and LASA metrics on this residue-by-residue basis is a gross oversimplification of the data we have generated. Supplementary Tables S1–S5 list these data on a cluster-by-cluster basis for: the soluble residue dataset (Table S1), soluble domain of the membrane dataset (mS, Table S2), the core-facing residues of the membrane dataset (mC, Table S3), the lipid-facing residues (lipid interactions on) of the membrane dataset (mL, Table S4), and the lipid-facing residues (lipid interactions off) of the membrane dataset (mN, Table S5). For discussion, a very small subset of these data (~0.3%) is provided in Table 3 and Table 4, for the soluble proteins, the soluble domain of the membrane proteins (mS), and the core-facing residues of the membrane proteins (mC) (Table 3) and the mL and mN residue sets (Table 4). Data for one bin of residue data for five somewhat diverse residues, ALA (***c5***), ASP (***c5.300***), ILE (***c5.300***), PHE (***c5.300***), and THR (***c5.300***), are listed. (The ***c5*** chess square is in the α-helix region of the Ramachandran plot.) Note that the ASP maps were calculated at a pH that evenly divides the ASP residues into charged, deprotonated aspartates and neutral protonated aspartic acids using concepts and procedures described earlier [37].

It is clear from Table 3 and Table 4 that the SASA/LASA values are not monotonic from residue to residue, or even within the same residue and chess square. They are, in fact, a distinguishing feature of each cluster. However, we showed earlier that the clustered maps can be numerically compared with similarity metrics and that there can be very similar 3D map motifs generated in different chess squares for the same residue, e.g., for ALA [35], or between different datasets, e.g., soluble proteins and soluble domains of membrane proteins [39]. We are not going to repeat that exercise here, but do highlight that ALA ***c5* 3020** and ALAmS ***c5* 905** were shown to have similar profiles, and as expected, their SASA values are very nearly the same. Also described earlier is that there is a degree of similarity between the core-facing membrane residue cluster maps (mC) and those of the soluble set [39], but the significantly smaller sample size of the former (~30-fold) is a confounding factor. The clusters of core-facing residue interaction maps show a similar diversity of SASAs as other cluster sets, but are generally more buried probably due to the confined space available in that environment.

Table 4 focuses on the mL vs. mN results, where the former has interactions with both other protein residues and the lipids, and thus has both LASA and SASA data, while the latter has only interactions with the protein (i.e., ignoring the lipid) and has only SASA data. Not surprisingly, the residues in this environment generally have much more significant propensity for interaction with lipids (LASA) than they do with H_2_O (SASA). However, this varies by residue type, chess square and cluster. For example, in ALA, the cluster **518** shows about 56% SASA, while **18** shows about 13%. Of the five residue types highlighted here, ASP has three clusters with higher SASA than LASA (**11**, **72** and **98**), while ILE and PHE have none. While these results are fairly intuitive based on the general expectations of residue properties, the next section enumerates the actual average interaction character of each cluster.

### 2.4. Hydropathic Interaction Characters

The HINT formalism considers four interaction types: favorable polar, Pol(+), such as acid–base and hydrogen bonding; unfavorable polar, Pol(−), such as acid–acid and base–base; favorable hydrophobic, Hyd(+); and unfavorable hydrophobic, Hyd(−), i.e., hydrophobic-polar representing desolvation and related effects [32,43]. Each individual residue’s interaction map set was analyzed by summing its grid point values by interaction type; these are recorded as the interaction map character [44]. Table 5 reports these on a residue type basis for all five datasets. For the purposes of clarity and to aid possible comparisons between the residues, the data in Table 5 were normalized by the random coil SASA dataset out in Table 2. These results are largely as expected, with hydrophobic residue sidechains being dominated by hydrophobic interactions, with the tendency for larger sidechains to have a larger fraction of their interactions to be favorable. The most productive residues for favorable polar interactions are the formally charged acids (ASP and GLU), amides (ASN and GLN), ARG (again, formally charged), and SER. GLY is an outlier because its data throughout this study are for the entire residue structure, not just the sidechain. As above for the SASA and LASA data, Supplementary Tables S1–S5 contain the interaction character data at the chess square and cluster levels (but are not normalized). Table 6 lists the (normalized) interaction character for the same set of residues in the soluble and mS datasets described in Table 3. Table 7 lists the (normalized) interaction character for the same set of residues in the mL and mN datasets described in Table 4.

Comparisons between the SASA/LASA data and interaction character data (e.g., Table 3 vs. Table 6 and Table 4 vs. Table 7) should be particularly informative. First, residue clusters with a high SASA + LASA make fewer measurable interactions (|Hyd(−)| + |Hyd(+)| + |Pol(−)| + |Pol(+)|), although interactions with water are incompletely measured: not all crystal structures in the soluble dataset have confirmed explicit waters. Second, even polar residues with a high LASA can have significant, often favorable, interactions with the DPPC lipid; see, for example, the ASP clusters **44** and **101** that have ~25–50% higher favorable polar interactions in the mL calculation than in the mN calculation. The THR clusters **6**, **33** and **55** have similar trends. Such residues must be interacting with the lipid’s polar head groups. Third, as expected, hydrophobic residues, especially ILE in Table 4 and Table 7, make significant hydrophobic interactions with the lipid in clusters with a high LASA, but the relationship is multifactorial. 

The key point is that the 3D interaction maps that we generated in our study, 14,158 for the soluble proteins and a total of 19,802 for the three subsets of the membrane proteins, are packed with information about the interaction preferences of the residues by type, backbone angle, and location. For those that are membrane-bound, we should have a handle on residue–lipid interactions as well as residue–residue interactions. Probing these maps by SASA/LASA or interaction character is inadequate to assess them.

### 2.5. Visualization of 3D Residue Interaction Maps

We selected a few maps, from the mL and mN datasets, for each of the residue sets listed in Table 4 and Table 7 for display in isovalued contour surfaces. Also shown are difference maps calculated between the cluster maps in the mL and mN datasets. Each map is presented in two side-by-side orientations. The orientation on the left has the z-axis (generally the CA–CB) bond directed out of the plane of the page, while the orientation on the right has the z-axis in the plane of the page directed to the right. The contour levels shown are as follows: red (opaque, –24, translucent, –6), blue (opaque, +24, translucent, +6), purple (opaque, –24, translucent, –6), and green (opaque, +12, translucent, +3,—except for PHE, where this contour level is +1) for unfavorable polar, favorable polar, unfavorable hydrophobic, and favorable hydrophobic interactions, respectively. 

#### 2.5.1. Alanine

**Figure 1:**
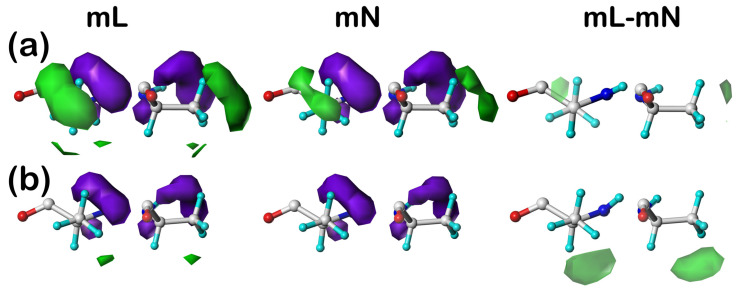
Three-dimensional clustered hydropathic interaction maps for the sidechains of the lipid-facing alanines in the ***c5*** chess square for (**a**) cluster 18 and (**b**) cluster 393. From the left, the first pair (mL) are the maps for interactions between alanine and all neighboring species, including the DPPC artificial lipids; the second pair (mN) are the maps for interactions between alanine and all neighboring species, excluding lipids; the third pair are the difference (mL–mN) maps, which thus characterize residue–lipid interactions. Each residue/map in a pair is displayed in two orientations: left—z-axis (CA-CB bond) directed out of the page, and right—z-axis directed upwards. Atoms are colored: C—white, H—cyan, N—blue, O—red. Green contours represent favorable hydrophobic interactions between the residue sidechain and its environment; purple contours represent unfavorable hydrophobic interactions.

In Figure 1 are plotted the clusters **18** (a) and **393** (b) in the ***c5*** chess square for ALAmL, ALAmN, and their ALAmL–ALAmN difference maps. To review, mL maps include the inter-residue and residue–lipid interactions and mN maps include only the inter-residue interactions, and thus the difference maps illustrate the role of lipid interactions. As it can be inferred from the close numerical match in the interaction characters (Table 7) of all ALAmL and ALAmN residues, alanine does not appear to make much of an impact in terms of protein–lipid interactions (at least in this conformation), which can be visualized in Figure 1 as the similarity of the analogous mL and mN maps (green and purple contours, favorable and unfavorable hydrophobic, respectively) and the very weak, but favorable, hydrophobic interactions seen in the difference maps. Alanine is simply too small to reach and associate with the membrane lipid molecules. It is worth noting, however, that the small patches of hydrophobic interaction density in the difference maps are at different locations (~10 o’clock in **18** and ~5 o’clock in **393**), thus showing diversity even in alanine’s small interaction with lipids.

#### 2.5.2. Aspartic Acid

**Figure 2:**
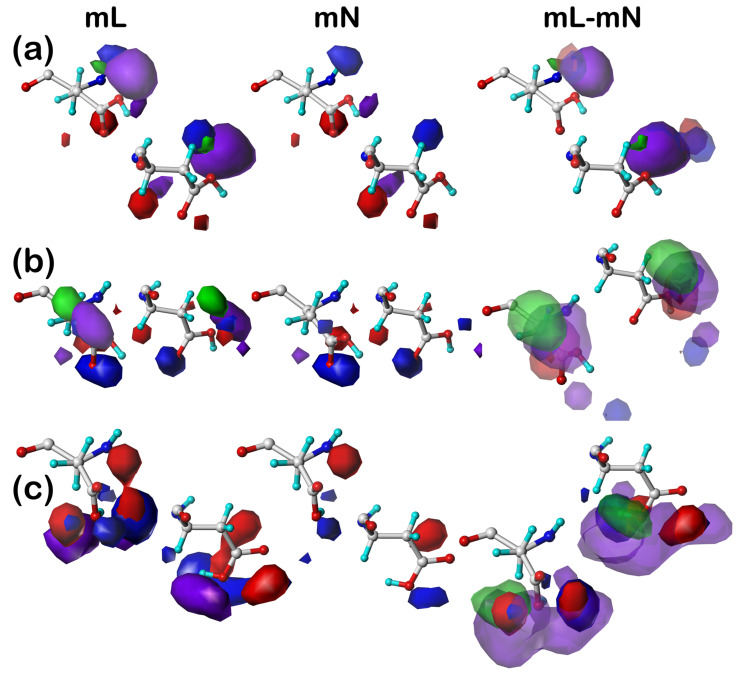
Three-dimensional clustered hydropathic interaction maps for the sidechains of the lipid-facing aspartic acids/aspartates in the ***c5*** chess square for (**a**) cluster **44**, (**b**) cluster **82**, and (**c**) cluster **101**. From the left, the first pair (mL) are the maps for interactions between aspartic acid/aspartate and all neighboring species, including the DPPC artificial lipids; the second pair (mN) are the maps for interactions between aspartic acid/aspartate and all neighboring species, excluding lipids; the third pair are the difference (mL–mN) maps, which thus characterize residue–lipid interactions. Each residue/map in a pair is displayed in two orientations: left—z-axis (CA-CB bond) directed out of the page, and right—z-axis directed upwards. Atoms are colored: C—white, H—cyan, N—blue, O—red. Green contours represent favorable hydrophobic interactions between the residue sidechain and its environment; purple contours represent unfavorable hydrophobic interactions; blue contours represent favorable polar interactions; and red contours represent unfavorable polar interactions.

Figure 2 illustrates the residue clusters **44** (a), **82** (b), and **101** (c) for ASPmL, ASPmN, and ASPmL–ASPmN in the 300° parse of the ***c5*** chess square, i.e., ***c5.300***. Unsurprisingly, aspartate/aspartic acid residues are dominated by polar interactions (red and blue) and unfavorable hydrophobic (purple), although favorable interactions with the CB methylene are strongly evident in cluster **82**, weakly in **101**, and barely appear in **44**. All three ASPmL clusters were calculated by our algorithm [37] to be protonated, and thus neutral (less polar), in keeping with their expected interactions with the hydrophobic membrane lipids. All of the mN maps, where interactions with lipids are turned off, show the expected interactions of an aspartic acid sidechain, making a few hydrogen bonds (blue) and detecting a few polar (red) and hydrophobic (purple) clashes [37]. The mL maps, and by inference the difference maps, are much more feature rich, reflecting the interesting diversities of interactions. Clearly, unfavorable hydrophobic (purple) interactions will be important here as this very polar sidechain interacts with the nonpolar lipids, but there are a large number of new red and blue interaction contours, especially in cluster **101**. This likely is due to aspartic acids/aspartates, which are *not* exceedingly rare in the lipid-facing region, fulfilling a structural role by anchoring the protein to the lipids through interactions with the DPPC head groups (or analogous molecules in Nature).

#### 2.5.3. Isoleucine

**Figure 3:**
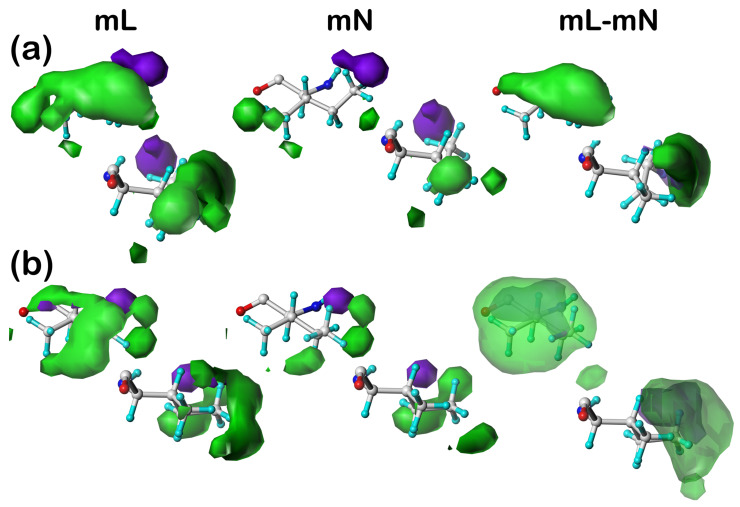
Three-dimensional clustered hydropathic interaction maps for the sidechains of the lipid-facing isoleucines in the ***c5*** chess square for (**a**) cluster **6** and (**b**) cluster **17**. From the left, the first pair (mL) are the maps for interactions between isoleucine and all neighboring species, including the DPPC artificial lipids; the second pair (mN) are the maps for interactions between isoleucine and all neighboring species, excluding lipids; the third pair are the difference (mL–mN) maps, which thus characterize residue–lipid interactions. Each residue/map in a pair is displayed in two orientations: left—z-axis (CA-CB bond) directed out of the page, and right—z-axis directed upwards. Atoms are colored: C—white, H—cyan, N—blue, O—red. Green contours represent favorable hydrophobic interactions between the residue sidechain and its environment; purple contours represent unfavorable hydrophobic interactions.

In Figure 3, the residues displayed are for the **6** (a) and **17** (b) map clusters of ILEmL, ILEmN, and ILEmL–ILEmN (***c5.300***). It is clear that isoleucine’s major impact on structure is by providing favorable hydrophobic interactions. The ILEmN clusters illustrate the interactions between this residue and its neighboring residues, i.e., it is playing a fairly similar role as alanine within the protein. The ILEmL maps are, however, completely dominated by interactions with the lipids, and the ILEm–ILEmN difference maps emphasize how important this residue is to structurally maintaining protein/lipid ensembles.

#### 2.5.4. Phenylalanine

**Figure 4:**
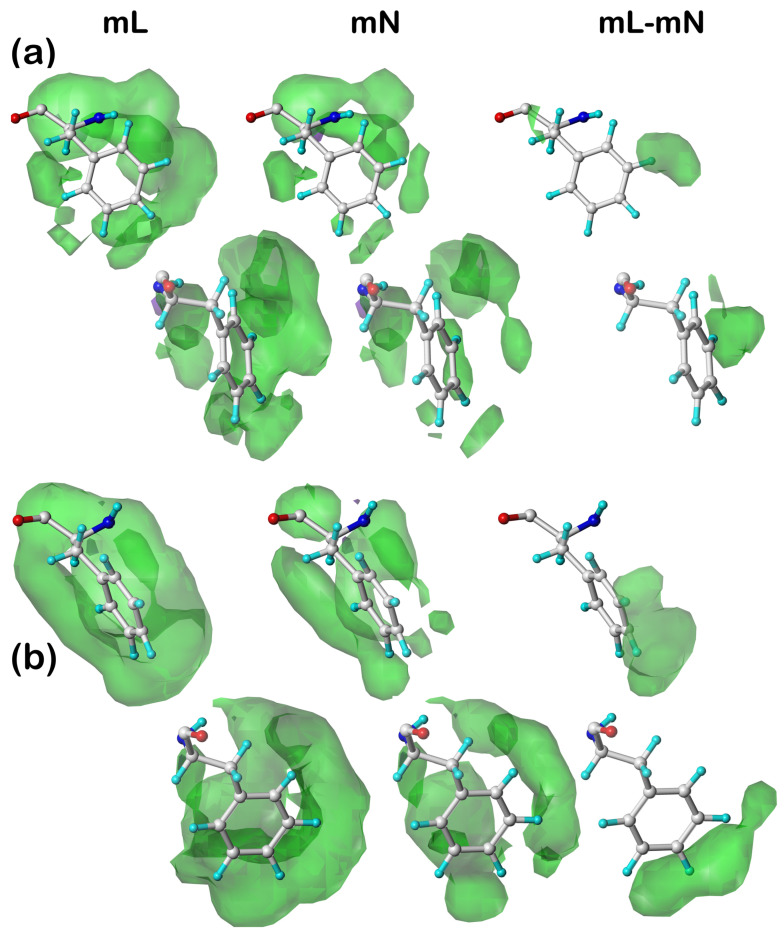
Three-dimensional clustered hydropathic interaction maps for the sidechains of the lipid-facing phenylalanines in the ***c5*** chess square for (**a**) cluster **110** and (**b**) cluster **202**. From the left, the first pair (mL) are the maps for interactions between phenylalanine and all neighboring species, including the DPPC artificial lipids; the second pair (mN) are the maps for interactions between phenylalanine and all neighboring species, excluding lipids; the third pair are the difference (mL–mN) maps, which thus characterize residue–lipid interactions. Each residue/map in a pair is displayed in two orientations: left—z-axis (CA-CB bond) directed out of the page, and right—z-axis directed upwards. Atoms are colored: C—white, H—cyan, N—blue, O—red. Green contours represent favorable hydrophobic interactions between the residue sidechain and its environment.

Figure 4 sets out residue sidechain cluster maps **110** (a) and **202** (b) for the phenylalanine datasets PHEmL, PHEmN and PHEmL–PHEmN (***c5.300***). Note that, although these two clusters show significantly different orientations of the aromatic ring (χ^2^ = 97° for residue 110 and χ^2^ = 151° for residue 202), the calculation and clustering protocol correctly binned these as distinct clusters possessing different conformations as well as unique interaction sets. Also, while there *are* significant favorable hydrophobic interactions produced by phenylalanine, they are spread out over a larger volume; so, as noted above, the favorable hydrophobic maps (green) are contoured at a lower value for just this figure. The HINT formalism considers π-π stacking as a favorable hydrophobic interaction [36,43]; so, the only interaction types that are significant are favorable hydrophobic. There is no indication from Table 5 or Table 7 that any different result would be expected in other clusters or chess squares in lipid-facing phenylalanine datasets. Of particular interest are the (PHEmL–PHEmN) difference maps: the two results shown here expose different edges to the lipids. Cluster **110** is exposing only one of its CE atoms, while cluster **202** is exposing a crescent of interaction potential spanning CE1–CZ–CE2. Both, however, are showing edge-on rather than stacking interactions.

#### 2.5.5. Threonine

**Figure 5:**
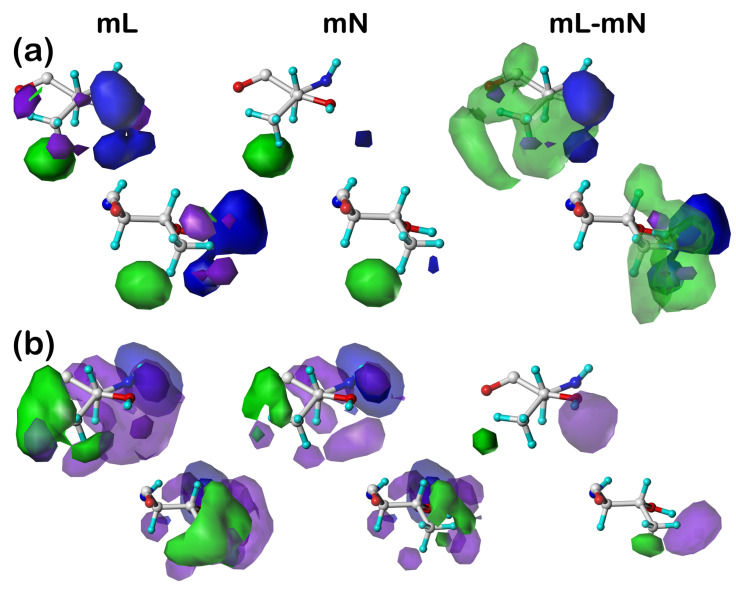
Three-dimensional clustered hydropathic interaction maps for the sidechains of the lipid-facing threonines in the ***c5*** chess square for (**a**) cluster **33** and (**b**) cluster **52**. From the left, the first pair (mL) are the maps for interactions between threonine and all neighboring species, including the DPPC artificial lipids; the second pair (mN) are the maps for interactions between threonine and all neighboring species, excluding lipids; the third pair are the difference (mL–mN) maps, which thus characterize residue–lipid interactions. Each residue/map in a pair is displayed in two orientations: left—z-axis (CA-CB bond) directed out of the page, and right—z-axis directed upwards. Atoms are colored: C—white, H—cyan, N—blue, O—red. Green contours represent favorable hydrophobic interactions between the residue sidechain and its environment; purple contours represent unfavorable hydrophobic interactions; and blue contours represent favorable polar interactions.

Two examples of threonine interaction cluster maps, **33** (a) and **52** (b), are displayed in Figure 5. In this case, the two clusters are quite different from each other. Cluster **33** makes significant favorable polar interactions with the lipid, associated with the OG1 atom, while its methyl (CG2) appears to be embedded within the hydrophobic portions of the lipid. In contrast, cluster **52**’s interactions with the lipid molecules are wholly hydrophobic, albeit both favorable and unfavorable. Profiles for the other four threonine cluster maps, **6**, **55**, **60,** and **71**, can be largely surmised from Table 4 and Table 7. Map **6** shows the largest difference in character (Table 7) for unfavorable hydrophobic, with more modest differences in favorable hydrophobic and polar, suggesting that the OG1 atom is interacting favorably with a polar atom in a DPPC and likely unfavorably with other atoms in it. These differences for cluster **55** are similar, except that of the favorable hydrophobic is somewhat larger than the unfavorable hydrophobic. Clusters **60** and **71** are different in that their overall interactions are weaker for all four types, as should be expected by the their substantially larger accessible surface areas—implying more empty space surrounding these clusters.

### 2.6. Composite 3D Interaction Maps for Proteins

With the tens of thousands of backbone-, residue-, and environment-dependent distinct map clusters generated for this study, we can assemble an overall picture of non-covalent interactions within a protein, between proteins, between proteins and ligands, and of special interest here, between proteins in transmembrane regions and their lipid support. For this discussion, we arbitrarily chose one protein/lipid structure from the training set, pdbid: 4o6y, which is the X-ray diffraction structure (resolution of 1.70 Å) of cytochrome b561 from *Arabidopsis thaliana*, a eukaryotic transmembrane ascorbate-dependent oxidoreductase [45]. 

**Figure 6:**
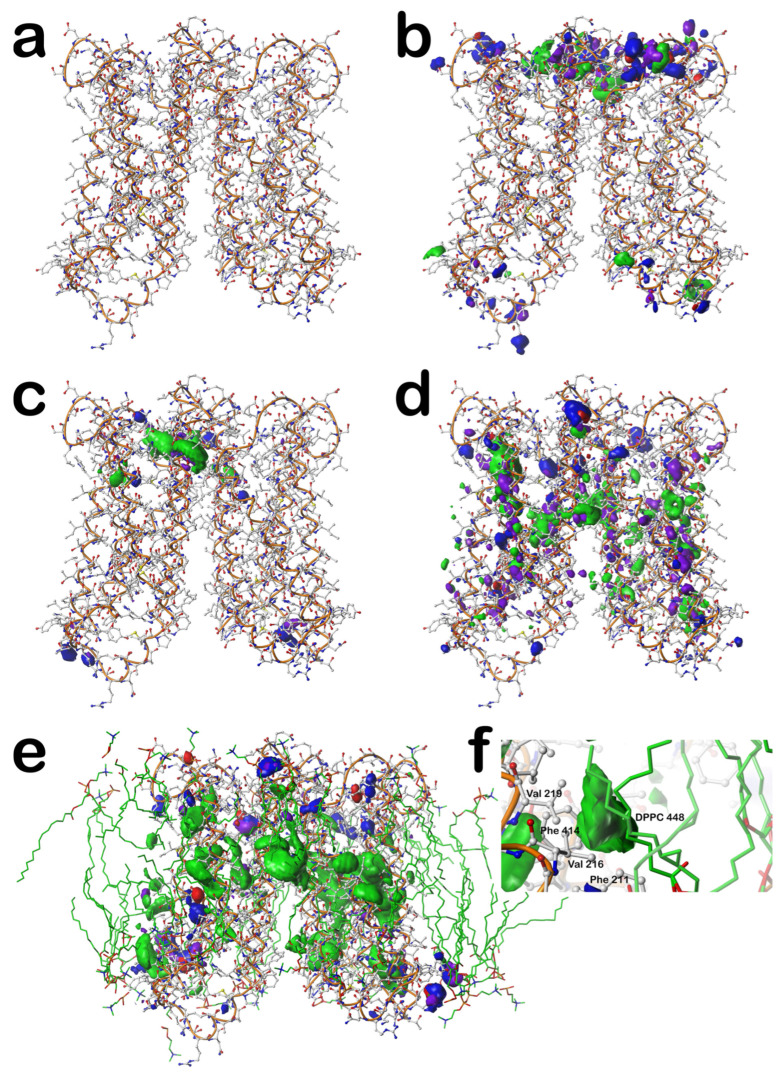
Structures and hydropathic interactions of cytochrome b561 from *Arabidopsis thaliana* (pdbid: 4o6y). (**a**) The hydrogen-suppressed molecular structure with superimposed tube (orange) illustrating the backbone trace. Atoms are colored by type (white = carbon). (**b**) Hydropathic interactions between each residue’s sidechain and environment for atoms in the mS soluble domains of the protein. Green contours represent favorable hydrophobic interactions between the residue sidechain and its environment; purple contours represent unfavorable hydrophobic interactions; blue contours represent favorable polar interactions; and red contours represent unfavorable polar interactions. (**c**) Hydropathic interactions between each residue’s sidechain and environment for the mC core-facing residues in the intracellular domain of the protein. Contour colors are as above. (**d**) Hydropathic interactions between each residue’s sidechain and environment (excluding protein–lipid interactions, i.e., mN dataset) for lipid-facing residues in the intracellular domain of the protein. Contour colors are as above. (**e**) Hydropathic interactions between each residue’s sidechain and lipid molecules (mL-mN) in the intracellular domain of the protein. Contour colors are as above. (**f**) Zoomed view of one specific interaction contour—between the protein residue sidechains of Phe 211, Val 216, Val 219, and Phe 414 and the lipid molecule DPPC 448.

We traced each residue in the protein through our calculational procedure to identify its membrane protein subset, chess square, parse, and cluster membership. Next, we constructed three-dimensional grids large enough to encage the entire protein, 99 × 95 × 111 for mC residues, 153 × 127 × 113 for mL residues, and 135 × 125 × 145 for mS residues, all with 0.5 Å grid spacing. Then, the 3D clustered interaction maps for each residue in the protein were frame-shifted to that residue’s frame, and the interpolated values for each grid point in the residue-level maps (for favorable and unfavorable, hydrophobic and polar interactions) were accumulated in the protein level maps. Figure 6 sets out these results.

First, in Figure 6a, the structure for the cytochrome b561 protein is shown; it has two chains, each possessing six transmembrane helices. There are a relatively small number of extramembrane residues, largely on the extracellular side (top, Figure 6a). Also, the gap between the two chains is noticeably smaller on the extracellular side than on the intracellular side. In Figure 6b, the composite interaction maps for the mS residues outside the membrane surfaces (extracellular on top and intracellular on the bottom) are shown (see caption for display details). As suggested by the relative number of extramembrane residues (top and bottom), the extracellular region has a much richer set of interactions than the intracellular. Each displayed contour corresponds to one or more interactions between the residue sidechains that impact the structure: the blue isovalue surfaces represent favorable polar interactions like hydrogen bonds, just as described above for the individual residue-level maps; red = unfavorable polar; green = favorable hydrophobic; and purple = unfavorable hydrophobic. 

In the transmembrane (helix) region, we first display the interactions between the core-facing mC residues and their neighbors in Figure 6c. It is important to point out that all of these map figures (Figure 1, Figure 2, Figure 3, Figure 4, Figure 5 and Figure 6) were manually contoured, i.e., the isovalues were chosen to maximize reader viewability and information accessibility. The digital information within the clustered map grids themselves is the key result. With that in mind, Figure 6c could be contoured such that every atom pair is associated with an interaction or that no interactions are shown at all. Thus, this figure shows that there are numerous favorable and relatively significant interactions at or near the junction between the two six-helical bundles. Of the 17 residues identified as mC in this protein, there are 2 ARGmC, 4 HISmC, 4 LYSmC, and 1 TRPmC, all very polar and large. More research is called for, beyond the scope of this work, but these results are suggestive that the mapping strategy may provide mechanistic insights into the structure/function in membrane proteins. For example, our pH-dependent map data may be particularly informative.

Figure 6d focuses on the “internal” residue–residue interactions between the lipid-facing residues, i.e., the mL dataset, excluding interactions with lipids, which is designated mN. It highlights the hydrogen bonds and favorable/unfavorable hydrophobic interactions and very few unfavorable polar interactions, which characterize this protein’s structure. Clearly, there are a large number of hydrogen bonds, and as should be expected since over half of the residues in mL are hydrophobic, a substantial volume of hydrophobic interactions is also seen. The prominent unfavorable hydrophobic, although technically an energetic cost, always tracks with the favorable hydrophobic due to interactions between hydrophobic sidechains and nearby polar residues or ubiquitous backbone atoms within the protein.

Shown in Figure 6e is the map for residue–lipid interactions, i.e., mL–mN. This figure also includes the molecular model of the associated DPPC lipids (with green-rendered carbons) extracted from the simulation reported in the MemProtMD database [40]. Unsurprisingly, the contours shown are dominated by those that are favorable hydrophobic, especially in the middle where the lipid tails are found. Clearly, however, the lipid headgroups are also recognized by the protein’s transmembrane helixes, and their sequence is quite obviously designed by Nature to anchor within the cellular membrane with both polar and hydrophobic interactions. There are literally thousands of specific protein–lipid interactions comprising this map. To illustrate the information content (and complexity) of this composite map, we isolated just one favorable hydrophobic contour in Figure 6f. This shows that the lipid tails of DPPC 448 (nomenclature from MemProtMD) interact with the hydrophobic sidechains of Phe 211, Val 216, Val 219, and Phe 414. 

While this protein’s analysis is of an existing structure, there clearly is very significant scope for using this information in structure prediction applications. Strategies for this are described in the next section.

### 2.7. Reconstruction of Protein Structure Using 3D Interaction Maps

The approach to reconstruct the maps into a structure prediction is simply to match the maps’ patterns such that as many structural interaction features within sidechains as possible are reinforced. Thus, as each individual residue interaction map encodes a specific set of interactions representing a plausible environment, the “ideal” interacting residue or residues would have map features with the same character at the same points in space when all residue maps are aligned in the “protein frame”. As was seen in many of the individual residue interaction maps (Figure 1, Figure 2, Figure 3, Figure 4 and Figure 5), as well as in the 4o6y composite map (Figure 6), this does not mean that all interactions in a stable protein structure are favorable. 

Most maps include several to a dozen distinct interaction features that are, in principle, at the midpoint between the two (or more) interacting functional groups or atoms. This suggests a surfeit of information for self-consistently reassembling the protein’s sidechain conformations. The advantage here over conventional technology, such as backbone-dependent rotamer libraries [46,47,48,49], and codes, such as SCWRL [50,51,52], is that this optimization is not just geometric, or including just hydrogen bonds, but inventories the entire hydropathic interaction network in its optimization. 

However, reconstruction remains a combinatorial problem, albeit a smaller one: (1) instead of a near infinite number of backbone conformations, our chessboard schema defines only sixty-four, of which, except for GLY, less than half are significantly populated; (2) instead of a near infinite number of sidechain conformations for most residues, we systematized each to an accessible number—between one (no χ^1^) and nine (χ^1^ and χ^2^), for each of which there are between four and eighteen maps; and (3) as the interaction map clusters are quite unevenly distributed amongst the residue types, backbone, and sidechain conformations, dictated by the populations of residues in the experimental structural data, consequently predicting structure for new cases will follow the same rules, i.e., the more common residues and conformations will have more extensive and validated training data than rare or potentially Ramachandran-disallowed backbone conformations. 

To proceed, 3D grids enclosing the protein or its region of interest can be constructed, much as described above for the creation of Figure 6. In this case, the grids will contain “scores” assessing the quality of interaction feature matches at the positions in space associated with each grid point. Encoding match scores as well as the molecular interaction features in 3D maps has operational computing advantages. They are easily parallelizable, perhaps even more than “embarrassingly” so, and are similarly completely amenable to GPU computing. Many popular scoring functions for docking use grid-based algorithms [53,54,55].

#### 2.7.1. Building a Structural Model from Interaction Maps

**Figure 7:**
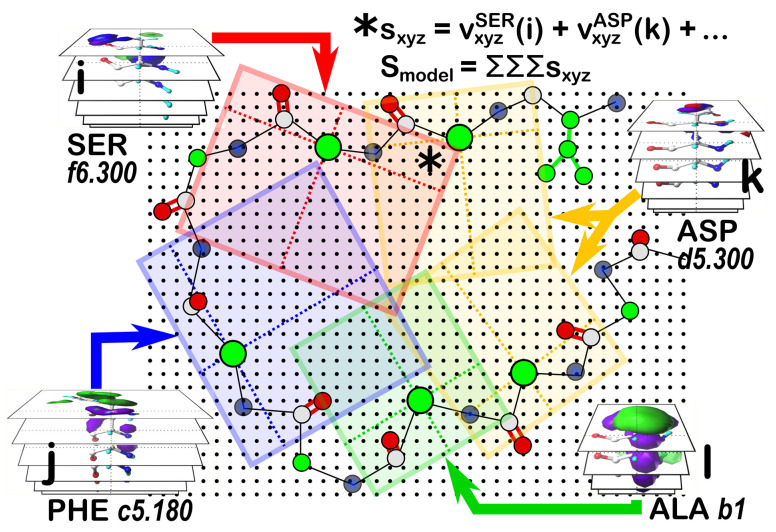
Cartoon depicting a method to optimize sidechain interactions and conformations. An overall score grid (black regular-spaced dots) is superimposed over the region of interest in the coordinate frame of the protein. Members (i, j, k, l) of the sidechain interaction map set for each residue are individually auditioned by frame-shifting onto the protein, followed by interpolation onto the score grid. Each point (xyz) in the score grid (*) is the sum (s_xyz_) of the values (v_xyz_) from each residue’s auditioned map. The total score for this model (S_model_) is then the sum over all grid points in the grid.

Applying our strategy for building a sidechain-optimized structural model by matching interaction features is illustrated by the cartoon of Figure 7. The score grid, similar to the map composite grid, holds at each grid point (xyz) the sum of all interaction maps from residues (i, j, k l, …) that have been frame-shifted to align with the protein’s backbone. The total score (S_model_) would be the sum of all values (s_xyz_) in the score grid. The difference between this score grid and the composite display maps is that S_model_ is a fitness function that must be optimized to define the best set of sidechain backbone conformations. Since SER has six maps per parse, PHE has twelve, each ASP has twelve, and ALA has four, the cartoon example suggests that there are 41,472 unique models to be scored, which is a problem amenable to various machine learning or evolutionary algorithm solutions, especially for the more biologically relevant cases of the tens-to-hundreds of residues to be modeled. We are working on a genetic algorithm solution to this problem [56].

#### 2.7.2. Underlying a Residue’s Molecular Structure

Each 3D interaction cluster map is associated with two molecular structures or coordinate sets: one being that of the specific residue responsible for the cluster exemplar and the other an average of all members of the cluster, weighted for the Euclidian distance from the cluster’s centroid [34]. For each cluster’s average structure, we have access to RMSDs for each of its atoms as well as average sidechain dihedral angles with standard deviations. Thus, depending on the scope of the problem and the quality of experimental data, the number of residue maps to be explored can be controlled. For example, if the sidechains are well resolved, the structural filters taking into account the now lesser uncertainties of atomic positions and dihedral angles can reduce the pool size of residue maps to be auditioned. In contrast, a poorly resolved structure or one missing a few or all sidechains might require auditioning larger sets of residue maps, e.g., the trial map set for serine may include all three χ^1^ parses.

#### 2.7.3. Fitting Lipids to a Membrane Protein Structure

A relatively small number of experimental membrane protein structures deposited in the protein data bank or other databases include any or more than a small handful of structural lipids in the transmembrane region, let alone their native set [19,20,57,58,59,60,61]. This experimental deficit has led to a number of proposed methodologies to simulate the lipid structures [62,63,64] or to predict or simulate protein structure within implicit [65,66,67,68] or explicit [69,70,71,72] membrane environments. 

The observation by Qin et al. [9] that there were conserved lipid binding sites on transmembrane proteins led us to believe that, just as the three-dimensional hydropathic interaction maps that we have been developing, and illustrated here, represent conserved interaction motifs possessing conserved character, loci, strength of interactions, etc., they may also include lipid interaction information if constructed from an appropriate training set. Furthermore, they would represent a means to develop an alternative, structure-based, approach for building reliable protein–lipid assemblies at a modest cost compared to many molecular dynamics-based approaches. 

Simply, referring back to Figure 6 and Section 2.7.1, if the lipid-facing amino acid residues in the transmembrane region are modeled and optimized with the mN dataset (similar, visually, to Figure 6d), their mL (or mL-mN, as in Figure 6e) analogues spotlight where hydrophobic, acid, base, etc. features on lipid molecules need to be placed to produce the indicated interactions indicated by the maps. 

## 3. Materials and Methods

### 3.1. Soluble Protein Dataset

As previously described, our dataset was comprised of 2703 randomly selected proteins from the RCSB Protein Data Bank, excluding those structures that contained ligand and/or cofactors [34]. Each residue type was extracted and studied independently from all structures, except N- and C-terminal residues. Selection criteria have been outlined previously [34], where we provided extensive documentation on our selection criteria for this protein library, but briefly: (1) We did not filter for redundancy, believing that more common motifs (and residue environments) should be sampled more in less in line with their population in Nature. This, of course, assumes that the PDB structures are themselves representative, which is probably only partly true; and (2) We did not filter for resolution, believing that many low-resolution structures have unique features and interaction motifs not present in the highest resolution PDB set. Hydrogen atoms were added as needed based on their hybridization, which was followed by conjugate the gradient minimizations of their positions using Sybyl X.2.1. No further structure optimizations were performed, as retaining the integrity of the experimental data was paramount. 

The presence or absence of water molecules in PDB structures is, nevertheless, a concern: we have commented on this issue extensively in the past [7,73,74,75], but there are few—only poor—options to rectify this after a structure is solved and deposited. However, our methods will usually highlight where water molecules (or other molecules playing a similar interaction role) should be. Unfulfilled polar interaction contours are clear indicators of a missing water molecule. The map clustering we performed does recognize hydrated and water-free residues as different, with distinct interaction profiles. It is important to note that our methods are silent regarding waters associated with the protein’s surface unless they make interactions.

Molecular models for four residue types—aspartic acid, cysteine, glutamic acid, and histidine—were built such that their ionization states fit pH levels previously determined (pH_50_) [37] to evenly divide each of those residues sets into protonated and deprotonated sets. The detailed procedure for this operation was reported previously [37,38]. Simply, the HINT interaction score between the residue of interest and its neighbors was calculated for both the protonated and deprotonated cases for ASP, CYS, and GLU (protonated cases were optimized for the highest scoring –XH angle) residues and for cases representing all eight [37] possible versions of HIS. Each of these scores were energetically corrected for pH—pK_a_^nominal^ and the case with the highest resulting HINT score defined the most likely ionization state for that residue. These pH_50_ values are 3.3449 for soluble ASP, 9.5814 for soluble CYS, 4.2240 for soluble GLU, and 5.1743 for soluble HIS. For all the membrane protein datasets, analogous pH_50_s were used: 3.9331 for ASP, 9.4506 for CYS, 4.2021 for GLU, and 5.4206 for HIS. Residues whose actual environment at the specified pH_50_ is basic were modeled as deprotonated and those in an acidic environment were modeled as protonated.

### 3.2. Membrane Protein Dataset

Similarly, we randomly extracted 362 membrane protein structures from the Grazhdankin et al. (2020) [76] dataset that is a subset of the MemProtMD database [40]. These structures were available as pre-oriented, lipid-“solvated” and optimized with ~1 μs of coarse-grain molecular dynamics [58]. In previous work [38,44], our dataset was slightly larger, but the Supplementary File (vide infra) for seven proteins (pdbids: 3fb5, 3wxv, 4xwk, 5f1c, 5jsz, 5llu, and 5m94) we used are not currently available. No effort was made to filter the membrane protein structures for redundancy or resolution. We removed lipids more than 6 Å away from the protein and added missing hydrogen atoms that were energy minimized as above. The MemProtMD dataset structures do not include water molecules, ions, or other cofactors. 

As described earlier, we further binned the residues into sets that described their locations within the membrane proteins [39]. Briefly, we adapted two of the Supplementary Files in MemProtMD found with each protein–lipid model. Snapshots of the average lipid phosphate surfaces (over the final 800 ns of simulation) are the “distortions“ coordinates (named *pdbid*_default_dppc-distortions.pdb) [40] that represent the membrane region’s extents. We thus defined the average z-coordinates as upper and lower planes and assigned all residues with all three of their backbone atoms, N, CA, and C, between those planes to be in the membrane region. Residues outside these planes were binned being in the (mS) soluble domain. Those in the membrane region were binned as lipid-facing (mL) or core-facing (mC) with the MemProtMD “residue-wise analysis” file (*pdbid*_default_dppc-by-resid.csv) that defines each residue to be a constituent (or not) of the pore inner surface. The mN datasets were identical to the mL datasets but the downstream map calculations were different (vide infra). 

### 3.3. Alignment Calculations

We applied a chessboard schema to identify secondary structure bins for all residues. This systematization yields sixty-four 45° by 45° backbone angle bins of the standard Ramachandran ϕ (phi)–ψ (psi) plot [34]. We calculated the ϕ, ψ, and χ angles for every residue in our datasets and binned each residue accordingly into their proper chess square based on the measured ϕ and ψ angles. All residues other than ALA, GLY, PRO, and VAL were further divided by their χ_1_ angles into three parse groups: group “***.60***”, (0° ≤ χ_1_ < 120°), group “***.180***” (120° ≤ χ_1_ < 240°), and group “***.300***” (240° ≤ χ_1_ < 360°). In the case of proline, the residues were parsed by their χ_1_ angles into two bins, –30° (330°) and +30°, which we denoted as “***.30m***” and “***.30p***”, respectively. Furthermore, ARG, GLN, GLU, and LYS were parsed also by χ_2_, again into “***.60***”, “***.180***”, and “***.300***” parse groups, i.e., these residues have nine 2-angle parses [37,39]. 

All individual residues (and their protein) were aligned to a stub residue at the center of each chess square, such that the Cartesian origin is at the CA atom, the CA-CB bond corresponds to the z-axis, and the CA-HA bond is in the yz-plane [34]. All calculated maps for that residue result from its interaction with its environment result from and can be aligned with and compared to all other residues of that type binned into that chess square.

To simplify nomenclature for this work, each residue was assigned a number in a list of residues within each chess square or, as needed, χ_1_ parse and χ_2_ parse, e.g., the first alanine in the ***a1*** chess square is 1, the third isoleucine in the ***c5.300*** parse is 3, etc. These codes can be unpacked using data freely available upon request from the author.

### 3.4. HINT Scoring Function 

Interatomic interactions were scored with the HINT forcefield [32,33]. The fundamental parameter, the hydrophobic atom constant, is an atom-level logP_o/w_ that uses the defined fragments and factors of the Hansch and Leo methodology [77,78]. These interactions were calculated for the residue of interest with respect to all other residue types and water, but for the mL lipid datasets, atoms from the DPPC lipid molecules were also considered in the calculations.

### 3.5. HINT Basis Interaction Maps

Three-dimensional boxes large enough to fit the structure of each residue type in all expected conformations, with an added 5 Å on each dimension, were calculated, all with a grid point spacing of 0.5 Å on each axis. Interaction maps illustrating in 3D all interactions of that residue—more or less a recasting of pairwise HINT scores into 3D objects—were calculated. These maps encode the position, intensity, and type of atom–atom interactions between the residue and other residues in contact or near-contact to it. Mathematical expressions have been set out in previous works [30,33,35]. Map data were calculated for sidechain atoms of all residues in this study with individual maps for the four interaction classes: favorable polar, unfavorable polar, favorable hydrophobic, and unfavorable hydrophobic.

### 3.6. Calculation of Map–Map Correlation Metrics and Clustering

The calculations of map–map correlations, i.e., comparisons of map pairs, was performed using an algorithm that defines the similarity between them, as previously described in detail [30]. To cluster the pairwise map similarity matrices, we utilized k-means clustering available as a supplement to the freely available R programing language and environment [79]. We opted to set a uniform maximum number of clusters for each chess square or chess square/parse to improve consistency and facilitate comparisons, as described earlier [73]. One issue with k-means is that it does not form singleton clusters; we backfilled as necessary such missing (singleton) clusters. Only chess square/parses with five or more maps were clustered, and those with fewer were instead averaged to create a pseudo 1-cluster case. Each cluster is named for the cluster member closest to its centroid and cluster names are presented in bold, e.g., **123**.

To represent each cluster, average maps were calculated by Gaussian weighting (*w*) each map’s contribution based on its Euclidean distance from the cluster centroid, as described earlier [34]. This weighting was performed so that maps closer to the centroid contribute more to the average map. The “exemplar” is the residue datum closest to the centroid of each cluster.

### 3.7. Solvent-Accessible and Lipid-Accessible Surface Area Calculations

The GETAREA algorithm [41] was used to calculate the solvent-accessible surface areas (SASAs) for all residue sidechains in our datasets. The program on its on-line server was run with default settings. For residues in the mC and mL datasets, the calculated SASAs were due to contact with water (both explicit and presumed) and with lipids. To quantify this latter effect, we calculated the ratio of the score sums involving lipid atoms to all atoms. If that ratio was greater than 0.1, we defined the resulting surface areas for such residues as LASAs or lipid-accessible surface areas. 

### 3.8. Creation of Composite and Score Maps

The methods to build both of these maps are similar. First, the region of interest is defined by calculating the minimum and maximum x, y, and z coordinates of the set of residues and lipids (if included); the maxima are increased by 5 Å, and the minima are decreased by 5 Å. A grid box is superimposed over that volume with spacing between grid points of 0.5 Å. For creating these final maps, the following temporary grids are created using this grid box: four floating point grid maps for each of the interaction types (favorable and unfavorable, hydrophobic, and polar) to hold the composite map data; one Boolean (integer) grid map for each of the residues of interest in the dataset recording whether each point of the overall map is relevant for its associated residue; eight integer maps for each of the residues of interest in the dataset that hold map point indices relating data points in the residue’s map to the overall map; and eight floating point grid maps for each of the residues of interest in the dataset holding the interpolation rules (values) for the alignment between the overall protein coordinate frame and each residue of interest’s coordinate frame. 

Each of these residues in the protein are aligned to their centroids, as presented above in Section 3.3 and previously [34]. The alignment matrix thus calculated is “unapplied” to the standard defined grid box associated with that residue type, which has now been expanded to explicitly enumerate all grid point coordinates in the box, rather than only the default eight corners. This positions the residue’s cluster maps in frame with the protein. For each point in the overall map that is encaged by eight grid points in the rotated and expanded residue map, interpolation rules are defined for that point by calculating the associated eight distances to the residue map’s grid point, normalizing them to the volume of the 8-point grid cube—in this case, 0.5^3^ = 0.125—and then recording those values in the eight interpolation rules grids for that specific protein residue. This pre-calculation, which is CPU- and memory-intensive, enables the very efficient population of the composite or numerous score maps downstream, without further rotations or interpolations.

## 4. Summary and Conclusions 

In this study, we reported the residue interaction environments of all amino acid residues previously undocumented in earlier articles [36,37,38,39]. One objective was to characterize residues performing unique structural roles in membrane proteins based on a classification scheme inspired by concepts and parameters available in the MemProtMD database [40]. We defined three membrane protein residue sets: (1) in soluble domains, (2) transmembrane and lipid-facing, and (3) transmembrane and core-facing. Generally, the residue interaction profiles as seen in the three-dimension hydropathic interaction maps from soluble domains in membrane proteins are quite similar to those of the same residues in soluble proteins. 

Treating the three membrane protein datasets as unique provides scope for more nuanced analyses of specific structural features as well as broader interpretations of structure. An in toto protein structure is, in our approach, the composite sum of nodes in a three-dimensional network where each hydropathic interaction residue map is a puzzle piece. The puzzle pieces for reassembling lipid-facing residue structure in the helices are particularly easy to interpret in this paradigm: the favorable hydrophobic and favorable polar interactions dominate the transmembrane protein–lipid contact surface volume. On the other hand, the transmembrane core region is more complex and suffers from sparser data—there are closer to thirty-fold fewer residues sampled in this dataset than in the soluble protein set. More raw structural data may have to be sampled here. However, as mentioned above, and one important point included in two of our earlier manuscripts [37,38], our methods include the ability to vary pH, which could be particularly interesting in building an understanding of structure–function relationships for membrane proteins. 

The three-dimensional hydropathic interaction maps provide a method to *deconstruct* the protein + water + lipid structure into residue-based units that carry enough information to reassemble such structures. However, these maps add value over simple molecular mechanics or homology methods of building proteins because they: incorporate indirect structural effects like the pi-pi stacking and pi-cation interactions of aromatic residues [39], model the role of ionization states in structure for ionizable residues [37,38], and even have strong potential to discover, define, and characterize binding sites on protein surfaces, e.g., for lipids as discussed here, but likely for ligands or cofactors. We term our methods “3D interaction homology” because the maps are focused on the three-dimensional arrangement of interactions and their types, and not the specific neighboring residue types. Further work on this paradigm will likely involve algorithms for the intelligent reassembly of the residue-level puzzle pieces into the full structure.

## Figures and Tables

**Table 1:** Enumeration of the datasets.

Residue	Soluble Proteins		Membrane Proteins (mS)			Membrane Proteins (mC)			Membrane Proteins (mL)		
	Maps	Clusters	Maps	Clusters	Fraction ^a^	Maps	Clusters	Fraction ^a^	Maps	Clusters	Fraction ^a^
ALA	57,107	167	16,566	173	0.487	1942	85	0.057	15,480	132	0.455
ARG	35,745	1381	11,786	905	0.714	1147	214	0.069	3581	466	0.217
ASN	34,743	829	9678	623	0.676	1303	204	0.091	3344	350	0.233
ASP	42,720	884	12,332	729	0.784	1079	188	0.069	2318	337	0.147
CYS	5500	294	2052	256	0.484	194	45	0.046	1991	118	0.470
CYX	5237	330	871	172	0.942	--	--	0.000	54	25	0.058
GLN	28,773	1275	8895	878	0.713	916	201	0.073	2664	387	0.214
GLU	49,301	1508	13,850	1097	0.795	1090	247	0.063	2487	395	0.143
GLY	54,484	487	14,668	427	0.504	2082	211	0.071	12,373	311	0.425
HIS	15,282	590	4422	432	0.647	543	101	0.079	1870	224	0.274
ILE	43,935	464	11,248	372	0.410	1800	148	0.066	14,405	307	0.525
LEU	69,013	561	19,450	510	0.427	2774	186	0.061	23,327	415	0.512
LYS	45,462	1639	11,639	1042	0.754	920	193	0.060	2878	415	0.186
MET	14,953	548	4470	370	0.431	825	117	0.080	5080	245	0.490
PHE	30,959	608	8117	476	0.360	1591	165	0.071	12,808	415	0.569
PRO	33,531	205	10,323	182	0.641	917	79	0.057	4861	142	0.302
SER	46,869	610	13,353	523	0.569	2002	221	0.085	8124	345	0.346
THR	41,424	466	11,611	432	0.544	1780	178	0.083	7950	294	0.373
TRP	11,636	458	2969	313	0.381	551	91	0.071	4271	272	0.548
TYR	28,889	609	7806	447	0.525	1317	156	0.089	5740	314	0.386
VAL	53,826	245	14,014	209	0.454	1879	99	0.061	14,992	165	0.485
TOTAL	749,389	14,158	210,120	10,599	0.531	26,652	3129	0.071	150,598	6074	0.399

^a^ Fraction of membrane protein residues found in the particular subset, i.e., in soluble domain (mS), within membrane and core-facing (mC), or within membrane and lipid-facing (mL).

**Table 2:** Solvent-accessible ^a^ and lipid-accessible ^b^ surface areas (SASA and LASA, respectively) of the datasets [all surface areas (Å^2^)].

Residue	Random Coil ^c^	Soluble	Membrane (mS)	Membrane (mC)			Membrane (mL)		
	SASA	SASA	SASA	ASA ^d^	SASA	LASA	ASA ^e^	SASA	LASA
ALA	64.9	17.0	25.5	12.3	11.5	0.8	13.3	3.2	10.1
ARG	195.5	82.9	36.6	37.4	35.9	1.5	75.6	26.4	49.5
ASN	114.3	48.9	37.5	25.6	24.0	1.6	28.8	11.5	17.3
ASP	113.0	49.9	41.8	26.3	25.0	1.3	35.0	16.1	18.9
CYS	102.3	9.2	10.9	9.6	8.9	0.7	17.9	5.7	12.2
CYX	(153.5) ^f^	--	--	--	--	--	--	--	--
GLN	143.7	62.2	38.1	29.6	27.9	1.7	39.2	19.0	20.2
GLU	141.2	68.4	45.5	34.3	33.0	1.3	39.7	20.4	19.4
GLY	(87.2) ^g^	0.0	0.0	0.0	0.0	0.0	0.0	0.0	0.0
HIS	154.6	49.0	32.3	36.3	30.0	6.3	47.6	15.6	32.0
ILE	147.3	17.6	13.6	21.0	18.3	2.7	41.8	4.7	37.1
LEU	146.2	20.0	16.1	21.3	19.0	2.3	43.7	4.8	38.8
LYS	164.5	89.8	50.1	47.8	44.8	3.0	78.3	26.3	53.2
MET	158.3	27.2	16.1	22.9	19.8	3.1	31.4	6.2	25.1
PHE	180.1	22.1	15.2	22.6	19.2	3.4	49.5	7.7	41.8
PRO	105.2	45.5	37.1	21.8	21.0	0.8	34.6	7.8	26.7
SER	77.4	29.0	33.5	16.1	15.1	1.0	16.0	5.4	10.5
THR	106.2	36.3	30.1	19.8	18.3	1.4	23.2	5.9	17.3
TRP	224.6	34.1	19.9	33.1	29.0	4.1	75.0	10.3	64.8
TYR	193.1	36.9	18.7	25.0	22.9	2.1	49.9	9.8	40.1
VAL	122.3	18.0	15.3	19.0	17.4	1.6	33.5	5.0	28.5

^a^ From GETAREA [41]; ^b^ Adapted from GETAREA results as described in the text; ^c^ Values provided by Fraczkiewicz and Braun [41] as surface areas for fully exposed residue sidechains; ^d^ Accessible surface area is the sum of SASA and LASA; ^e^ Accessible surface area is the same as SASA for the mN datasets; ^f^ GETAREA does not recognize S-S bridged cystines coded as CYX: this value is 150% of CYS random coil SASA; ^g^ GLY has no sidechain: this value is the random coil SASA for the full glycine residue.

**Table 3:** Solvent-accessible and lipid-accessible surface areas (SASA and LASA, respectively) for soluble proteins, mS, and mC datasets in selected residues/chess squares/χ^1^ parses [all surface areas (Å^2^)].

Residue	*Chess Square.Parse* ^a^	Soluble Set			Membrane Set (mS)			Membrane Set (mC)			
		Cluster ^b^	Count ^c^	SASA ^d^	Cluster ^b^	Count ^c^	SASA ^d^	Cluster ^b^	Count ^c^	SASA ^d^	LASA ^e^
ALA	***c5***	**829**	1245	42 ± 14	**128**	276	47 ± 25	**7**	69	2 ± 3	0 ± 4
		**1830**	578	61 ± 12	**771**	171	79 ± 26	**57**	32	12 ± 11	0 ± 0
		**3020**	1170	8 ± 11	**905**	555	7 ± 13	**86**	13	30 ± 14	3 ± 12
		**3449**	1645	11 ± 11	**996**	223	22 ± 25	**139**	43	13 ± 10	1 ± 7
ASP	***c5.300***	**217**	461	20 ± 18	**2**	59	53 ± 25	**8**	5	41 ± 17	0 ± 0
		**433**	255	92 ± 17	**70**	92	42 ± 15	**10**	6	12 ± 9	0 ± 0
		**747**	310	76 ± 24	**80**	67	28 ± 20	**17**	5	22 ± 12	0 ± 0
		**842**	173	107 ± 11	**126**	42	60 ± 20	**20**	5	52 ± 38	10 ± 20
		**990**	305	69 ± 22	**202**	51	62 ± 17	**22**	5	17 ± 8	0 ± 0
		**1093**	265	53 ± 25	**231**	77	62 ± 24	**26**	4	30 ± 15	0 ± 0
		**2394**	149	68 ± 20	**276**	133	16 ± 15	**33**	5	13 ± 3	0 ± 0
		**2452**	354	44 ± 18	**290**	48	26 ± 30	**38**	3	35 ± 8	0 ± 0
		**3069**	274	70 ± 18	**423**	39	58 ± 24				
		**3105**	134	93 ± 21	**490**	43	92 ± 11				
		**3280**	291	89 ± 20	**528**	55	69 ± 19				
		**3331**	438	31 ± 25	**576**	81	89 ± 14				
ILE	***c5.300***	**19**	17	40 ± 24	**14**	12	13 ± 12	**4**	6	19 ± 8	0 ± 0
		**26**	18	98 ± 18	**27**	11	67 ± 27	**8**	3	4 ± 3	0 ± 0
		**34**	36	7 ± 10	**30**	10	55 ± 5	**17**	5	5 ± 5	0 ± 0
		**38**	10	38 ± 19	**47**	15	11 ± 16	**18**	4	4 ± 7	15 ± 27
		**42**	35	3 ± 4	**55**	9	30 ± 26	**22**	4	4 ± 3	0 ± 0
		**100**	18	10 ± 10	**67**	15	25 ± 22	**23**	3	13 ± 9	7 ± 10
		**132**	14	130 ± 20	**71**	1	100 ± 0				
		**140**	15	42 ± 21	**88**	9	23 ± 26				
		**147**	15	22 ± 17	**90**	23	66 ± 3				
PHE	***c5.300***	**292**	186	28 ± 22	**29**	47	25 ± 22	**3**	4	8 ± 13	17 ± 17
		**451**	33	158 ± 61	**155**	47	9 ± 15	**9**	3	13 ± 3	0 ± 0
		**481**	276	10 ± 18	**233**	53	26 ± 19	**20**	11	10 ± 17	0 ± 0
		**529**	172	76 ± 38	**239**	3	85 ± 15	**22**	10	66 ± 49	27 ± 47
		**677**	60	103 ± 54	**252**	49	16 ± 18	**66**	20	10 ± 9	4 ± 16
		**1008**	188	13 ± 23	**394**	49	7 ± 13	**69**	16	30 ± 32	0 ± 0
		**1323**	190	30 ± 27	**398**	37	28 ± 24	**77**	2	47 ± 0	48 ± 0
		**1350**	92	100 ± 39	**408**	32	53 ± 24	**83**	4	13 ± 11	0 ± 0
		**1622**	214	10 ± 13	**409**	70	5 ± 11	**99**	31	15 ± 17	1 ± 5
		**1733**	184	31 ± 20	**411**	65	11 ± 13				
		**1837**	249	12 ± 15	**419**	59	17 ± 20				
		**1859**	241	6 ± 13	**508**	77	12 ± 18				
THR	***c5.300***	**23**	34	53 ± 30	**7**	11	52 ± 10	**9**	4	4 ± 2	0 ± 0
		**76**	34	83 ± 15	**11**	31	14 ± 26	**11**	3	61 ± 5	0 ± 0
		**110**	63	38 ± 20	**22**	14	34 ± 14	**12**	9	6 ± 6	0 ± 0
		**162**	14	98 ± 14	**38**	28	33 ± 27	**13**	2	4 ± 0	0 ± 0
		**209**	58	12 ± 15	**40**	11	94 ± 7				
		**220**	26	64 ± 13	**117**	22	76 ± 11				

^a^***Chess square.parse*** notation describes the residue’s backbone angle and χ^1^ bin (see text); ^b^ **Clusters** are named for the exemplar residue, i.e., closest to cluster centroid; ^c^ Number of residues of that type member of a specified cluster; ^d^ From GETAREA [41]; ^e^ Adapted from GETAREA results as described in text.

**Table 4:** Solvent-accessible and lipid-accessible surface areas (SASA and LASA, respectively) for mL/mN datasets in selected residues/chess squares/χ_1_ parses [all surface areas (Å^2^)].

Residue	*Chess Square.Parse* ^a^	Cluster ^b^	Member Count ^c^	Membrane (mL)		(mN)
				SASA ^d^	LASA ^e^	SASA ^d^
ALA	***c5***	**18**	307	1 ± 2	7 ± 14	8 ± 14
		**393**	172	3 ± 7	11 ± 19	15 ± 18
		**518**	62	18 ± 20	14 ± 23	32 ± 21
		**679**	158	4 ± 7	7 ± 15	11 ± 15
ASP	***c5.300***	**11**	6	57 ± 44	32 ± 45	78 ± 32
		**21**	17	4 ± 5	8 ± 16	12 ± 15
		**44**	8	0 ± 0	88 ± 21	88 ± 21
		**61**	17	4 ± 4	7 ± 17	10 ± 16
		**62**	8	5 ± 6	23 ± 29	29 ± 25
		**72**	9	18 ± 17	4 ± 10	21 ± 16
		**82**	8	7 ± 11	46 ± 36	53 ± 29
		**90**	9	23 ± 20	26 ± 47	49 ± 24
		**98**	13	42 ± 23	12 ± 29	54 ± 19
		**101**	6	15 ± 23	43 ± 32	59 ± 15
ILE	***c5.300***	**6**	8	9 ± 15	69 ± 48	77 ± 37
		**13**	16	0 ± 0	81 ± 53	82 ± 53
		**17**	22	3 ± 5	44 ± 40	47 ± 37
		**22**	27	2 ± 9	27 ± 36	29 ± 36
		**23**	14	6 ± 9	23 ± 34	27 ± 31
		**26**	20	0 ± 0	61 ± 34	61 ± 34
		**67**	29	4 ± 8	38 ± 44	42 ± 41
		**125**	13	10 ± 15	31 ± 36	41 ± 30
		**141**	8	21 ± 30	46 ± 45	67 ± 32
PHE	***c5.300***	**110**	54	4 ± 10	77 ± 58	82 ± 53
		**145**	72	9 ± 14	39 ± 53	49 ± 48
		**167**	47	19 ± 35	64 ± 56	83 ± 43
		**172**	100	9 ± 18	44 ± 44	54 ± 38
		**202**	59	8 ± 20	80 ± 53	88 ± 44
		**219**	3	0 ± 0	116 ± 27	116 ± 27
		**264**	85	3 ± 10	65 ± 55	68 ± 52
		**286**	85	7 ± 15	40 ± 46	46 ± 43
		**355**	22	22 ± 42	74 ± 63	97 ± 49
		**634**	66	9 ± 17	40 ± 42	49 ± 37
		**715**	100	7 ± 13	33 ± 43	39 ± 40
		**753**	82	5 ± 11	48 ± 47	53 ± 44
THR	***c5.300***	**6**	15	5 ± 6	26 ± 34	30 ± 31
		**33**	10	1 ± 3	41 ± 41	42 ± 40
		**52**	26	1 ± 4	28 ± 26	29 ± 24
		**55**	24	1 ± 1	15 ± 24	16 ± 23
		**60**	3	51 ± 37	33 ± 47	83 ± 14
		**71**	5	10 ± 21	63 ± 34	74 ± 16

^a^***Chess square.parse*** notation describes the residue’s backbone angle and χ_1_ bin (see text); ^b^ **Clusters** are named for exemplar residue, i.e., closest to cluster centroid; ^c^ Number of residues of that type member of a specified cluster; ^d^ From GETAREA [41]; ^e^ Adapted from GETAREA results as described in the text.

**Table 5:** Average interaction character ^a^ for the datasets by the residue type.

Res	Soluble				Membrane (S)				Membrane (C)				Membrane (L)				Membrane (N)			
	Hyd(−)	Hyd(+)	Pol(−)	Pol(+)	Hyd(−)	Hyd(+)	Pol(−)	Pol(+)	Hyd(−)	Hyd(+)	Pol(−)	Pol(+)	Hyd(−)	Hyd(+)	Pol(−)	Pol(+)	Hyd(−)	Hyd(+)	Pol(−)	Pol(+)
ALA	−158.6	45.9	---	---	−100.4	44.0	---	---	−105.8	45.3	---	---	−105.7	60.7	---	---	−100.9	48.2	---	---
ARG	−26.5	5.3	−37.8	93.9	−22.5	5.7	−9.7	66.7	−25.7	7.1	−13.5	76.4	−31.6	7.1	−10.8	81.3	−18.3	5.3	−10.0	52.3
ASN	−48.2	6.0	−86.8	122.7	−38.0	6.5	−40.4	60.8	−45.4	7.7	−45.3	65.8	−55.2	9.1	−50.9	74.3	−44.8	7.7	−43.1	64.2
ASP	−66.6	6.6	−176.6	287.9	−50.8	6.7	−57.7	107.3	−63.8	8.1	−71.9	143.1	−72.1	9.4	−72.4	129.6	−58.0	7.6	−66.2	127.9
CYS	−24.1	6.0	−26.7	58.1	−19.2	5.4	−17.9	42.3	−18.7	5.7	−13.7	47.5	−24.2	5.8	−12.0	42.3	−19.5	5.0	−11.7	40.4
CYX ^a^	−17.4	10.0	3.3	0.3	0.5	4.1	1.0	0.1	--	--	--	--	0.4	4.4	1.6	0.2	0.5	4.4	1.6	0.2
GLN	−47.3	7.5	−65.8	93.5	−35.0	8.0	−28.1	45.1	−41.5	10.3	33.6	52.6	−51.0	11.6	−39.5	60.5	−39.0	9.4	−30.6	49.6
GLU	−58.8	8.3	−111.3	179.9	−38.5	8.5	−29.9	58.8	−51.0	11.4	−41.3	85.5	−64.3	14.0	−46.5	87.9	−51.8	11.3	−40.4	85.0
GLY ^b^	−42.8	8.0	−103.0	143.7	−36.9	8.2	−59.5	86.8	−29.4	6.2	−46.9	69.7	−17.2	3.7	−25.3	37.8	−16.6	3.6	−25.1	40.1
HIS	−17.2	7.3	−43.9	51.5	−12.1	7.8	−20.2	22.6	−14.3	8.3	−21.0	24.0	−16.5	11.2	−20.7	24.2	−12.3	7.6	−16.8	20.1
ILE	−76.7	50.0	---	---	−54.6	46.1	---	---	−51.1	43.8	---	---	−44.1	55.4	---	---	−39.9	37.7	---	---
LEU	−79.6	50.0	---	---	−52.6	46.1	---	---	−48.8	44.9	---	---	−40.1	57.1	---	---	−35.7	38.2	---	---
LYS	−32.2	7.7	−16.1	42.4	−22.4	8.3	−3.4	31.7	−27.0	11.0	−8.1	43.0	−34.0	13.2	−4.2	47.7	−19.3	8.4	−4.1	25.9
MET	−19.7	13.6	−9.0	6.2	−14.0	13.5	−6.8	3.9	−14.0	−13.5	−6.5	3.7	−15.1	16.4	−6.0	3.1	−12.7	13.4	−5.1	2.9
PHE	−8.6	16.7	−10.6	6.4	−5.6	15.6	−1.2	4.4	−6.0	15.4	−1.0	3.9	−5.7	17.7	−0.6	3.2	−5.4	13.1	−0.4	2.5
PRO	−62.9	20.3	---	---	−34.7	20.9	---	---	−39.1	25.6	---	---	−39.3	29.2	--	--	−33.9	23.0	---	---
SER	−21.1	5.4	−45.1	103.2	−19.5	5.4	−18.5	66.5	−22.8	6.4	−20.7	66.7	−32.6	8.1	−19.8	69.7	−23.5	7.1	−16.2	11.7
THR	−87.0	20.8	−31.2	75.1	−54.2	21.4	−10.7	38.7	−56.1	26.1	−11.7	37.3	−59.9	34.5	−10.3	38.2	−50.8	26.9	−10.0	33.1
TRP	−8.9	14.4	−10.2	17.9	−6.5	13.4	−3.0	11.1	−6.9	13.9	−3.0	9.1	−8.0	15.3	−2.9	10.5	−6.1	10.6	−2.2	6.7
TYR	−18.8	12.3	−28.9	52.5	−16.7	11.9	−11.5	27.3	−18.0	12.6	−11.8	28.8	−22.5	13.0	−11.5	28.5	−15.7	10.7	−9.0	19.7
VAL	−95.3	48.0	---	---	−65.6	45.9	---	---	−63.2	43.0	---	---	−54.5	55.3	---	---	−50.6	39.2	---	---

^a^ Interaction character for a residue type is calculated as described in the text: Hyd(−) is unfavorable hydrophobic, Hyd(+) is favorable hydrophobic, Pol(−) is unfavorable polar, and Pol(+) is favorable polar. ^b^ Normalized by adjusted random coil SASA values described in Table 2 footnotes.

**Table 6:** Interaction character ^a^ by residue and cluster for the soluble and mS datasets.

Residue	*Chess Square.Parse* ^b^	Soluble Set					Membrane Set (mS)				
		Cluster ^c^	Hyd(−)	Hyd(+)	Pol(−)	Pol(+)	Cluster ^c^	Hyd(−)	Hyd(+)	Pol(−)	Pol(+)
ALA	***c5***	**829**	−164 ± 95	16 ± 6	--	--	**128**	−91 ± 30	21 ± 8	--	--
		**1830**	−117 ± 89	8 ± 4	--	--	**771**	−61 ± 26	11 ± 5	--	--
		**3020**	−164 ± 87	57 ± 21	--	--	**905**	−100 ± 28	60 ± 19	--	--
		**3449**	−166 ± 74	45 ± 17	--	--	**996**	−95 ± 36	41 ± 15	--	--
ASP	***c5.300***	**217**	−99 ± 35	12 ± 3	−245 ± 155	398 ± 231	**2**	−42 ± 17	7 ± 2	−45 ± 31	68 ± 68
		**433**	−20 ± 7	3 ± 1	−31 ± 19	32 ± 27	**70**	−38 ± 18	7 ± 2	−49 ± 26	75 ± 63
		**747**	−48 ± 20	3 ± 1	−183 ± 134	273 ± 203	**80**	−58 ± 21	5 ± 2	−85 ± 34	162 ± 96
		**842**	−12 ± 7	2 ± 1	−20 ± 14	18 ± 19	**126**	−27 ± 11	2 ± 1	−50 ± 26	83 ± 80
		**990**	−69 ± 25	4 ± 1	−281 ± 145	406 ± 221	**202**	−27 ± 14	4 ± 1	−34 ± 18	34 ± 23
		**1093**	−75 ± 33	5 ± 2	−237 ± 158	387 ± 317	**231**	−30 ± 10	5 ± 1	−32 ± 18	37 ± 37
		**2394**	−32 ± 18	6 ± 3	−35 ± 25	44 ± 47	**276**	−66 ± 26	11 ± 3	−85 ± 42	153 ± 105
		**2452**	−47 ± 17	8 ± 2	−88 ± 53	120 ± 83	**290**	−98 ± 30	14 ± 4	−73 ± 34	130 ± 90
		**3069**	−30 ± 12	5 ± 1	−48 ± 27	54 ± 40	**423**	−36 ± 20	4 ± 1	−48 ± 20	84 ± 59
		**3105**	−39 ± 16	2 ± 1	−159 ± 139	229 ± 205	**490**	−11 ± 6	1 ± 0	−21 ± 15	22 ± 37
		**3280**	−31 ± 10	3 ± 1	−55 ± 30	66 ± 46	**528**	−24 ± 9	3 ± 0	−43 ± 24	60 ± 54
		**3331**	−90 ± 27	9 ± 3	−334 ± 160	524 ± 222	**576**	−15 ± 7	2 ± 1	−18 ± 7	21 ± 31
ILE	***c5.300***	**19**	−77 ± 43	28 ± 9	--	--	**14**	−47 ± 13	38 ± 11	--	--
		**26**	−43 ± 25	12 ± 5	--	--	**27**	−43 ± 20	11 ± 4	--	--
		**34**	−76 ± 30	57 ± 12	--	--	**30**	−69 ± 22	49 ± 19	--	--
		**38**	−70 ± 37	41 ± 12	--	--	**47**	−50 ± 24	44 ± 10	--	--
		**42**	−53 ± 20	63 ± 12	--	--	**55**	−55 ± 28	23 ± 8	--	--
		**100**	−77 ± 46	54 ± 12	--	--	**67**	−73 ± 35	41 ± 9	--	--
		**132**	−50 ± 55	5 ± 2	--	--	**71**	−9 ± 0	1 ± 0	--	--
		**140**	−72 ± 55	44 ± 27	--	--	**88**	−49 ± 10	59 ± 14	--	--
		**147**	−73 ± 49	36 ± 14	--	--	**90**	−50 ± 18	57 ± 16	--	--
PHE	***c5.300***	**292**	−9 ± 3	14 ± 4	−6 ± 3	12 ± 4	**29**	−4 ± 1	14 ± 5	−0 ± 1	2 ± 1
		**451**	−8 ± 4	2 ± 1	−3 ± 6	4 ± 4	**155**	−4 ± 1	19 ± 4	−0 ± 1	3 ± 1
		**481**	−5 ± 1	21 ± 5	−0 ± 1	3 ± 1	**233**	−5 ± 2	9 ± 3	−1 ± 1	5 ± 2
		**529**	−6 ± 3	8 ± 3	−1 ± 1	4 ± 2	**239**	−4 ± 2	−48 ± 70	−0 ± 1	2 ± 1
		**677**	−4 ± 2	7 ± 5	−0 ± 0	2 ± 1	**252**	−5 ± 1	13 ± 3	−1 ± 1	5 ± 2
		**1008**	−6 ± 3	21 ± 6	−0 ± 1	3 ± 1	**394**	−5 ± 1	22 ± 3	−0 ± 0	3 ± 1
		**1323**	−13 ± 5	14 ± 4	−6 ± 6	12 ± 4	**398**	−6 ± 2	13 ± 4	−3 ± 2	8 ± 2
		**1350**	−12 ± 6	5 ± 2	−5 ± 3	9 ± 5	**408**	−5 ± 2	4 ± 1	−1 ± 1	4 ± 3
		**1622**	−12 ± 4	18 ± 4	−3 ± 2	8 ± 2	**409**	−4 ± 1	21 ± 4	−0 ± 1	3 ± 1
		**1733**	−8 ± 3	12 ± 4	−3 ± 2	7 ± 3	**411**	−5 ± 1	17 ± 4	−2 ± 2	6 ± 2
		**1837**	−6 ± 2	18 ± 5	−3 ± 2	7 ± 2	**419**	−5 ± 1	13 ± 4	−0 ± 1	3 ± 1
		**1859**	−9 ± 3	20 ± 5	−1 ± 1	4 ± 1	**508**	−6 ± 2	19 ± 5	−2 ± 1	6 ± 2
THR	***c5.300***	**23**	−71 ± 57	12 ± 8	−16 ± 14	46 ± 38	**7**	−40 ± 29	10 ± 3	−5 ± 4	34 ± 42
		**76**	−58 ± 38	4 ± 1	−31 ± 23	73 ± 53	**11**	−67 ± 22	29 ± 9	−17 ± 10	80 ± 62
		**110**	−91 ± 45	16 ± 6	−31 ± 15	89 ± 41	**22**	−35 ± 13	14 ± 3	−8 ± 5	25 ± 14
		**162**	−18 ± 7	3 ± 1	−7 ± 3	32 ± 24	**38**	−41 ± 17	20 ± 7	−7 ± 5	25 ± 15
		**209**	−89 ± 39	36 ± 11	−38 ± 24	86 ± 40	**40**	−9 ± 2	2 ± 1	−3 ± 5	7 ± 5
		**220**	−92 ± 55	10 ± 5	−37 ± 26	86 ± 45	**117**	−35 ± 23	4 ± 1	−4 ± 3	19 ± 19

^a^ Interaction character of a cluster is calculated as described in the text: Hyd(−) is unfavorable hydrophobic, Hyd(+) is favorable hydrophobic, Pol(−) is unfavorable polar, and Pol(+) is favorable polar; ^b^ ***Chess square.parse*** notation describes the residue’s backbone angle and χ_1_ bin (see text); ^c^ **Clusters** are named for the exemplar residue, i.e., closest to cluster centroid.

**Table 7:** Interaction character ^a^ by residue and cluster for the mL and mN datasets.

Residue	*Chess Square.Parse* ^b^	Cluster ^c^	Membrane Set (mL)				Membrane Set (mN)			
			Hyd(−)	Hyd(+)	Pol(−)	Pol(+)	Hyd(−)	Hyd(+)	Pol(−)	Pol(+)
ALA	***c5***	**18**	−93 ± 23	72 ± 20	--	--	−90 ± 22	59 ± 23	--	--
		**393**	−105 ± 41	57 ± 21	--	--	−88 ± 37	42 ± 19	--	--
		**518**	−91 ± 48	29 ± 28	--	--	−69 ± 30	21 ± 12	--	--
		**679**	−104 ± 30	54 ± 18	--	--	−95 ± 31	44 ± 17	--	--
ASP	***c5.300***	**11**	−24 ± 19	2 ± 1	−66 ± 56	83 ± 95	−23 ± 19	2 ± 1	−62 ± 58	80 ± 97
		**21**	−107 ± 31	14 ± 4	−100 ± 31	216 ± 59	−94 ± 30	12 ± 3	−86 ± 28	212 ± 62
		**44**	−115 ± 127	12 ± 4	−44 ± 34	47 ± 37	−22 ± 14	4 ± 2	−30 ± 30	24 ± 25
		**61**	−78 ± 21	9 ± 2	−120 ± 39	226 ± 137	−69 ± 21	9 ± 2	−110 ± 45	223 ± 138
		**62**	−94 ± 20	12 ± 3	−65 ± 23	100 ± 64	−70 ± 30	10 ± 4	−49 ± 33	98 ± 67
		**72**	−71 ± 30	7 ± 1	−105 ± 36	195 ± 120	−69 ± 31	7 ± 1	−105 ± 36	202 ± 126
		**82**	−63 ± 9	10 ± 4	−49 ± 25	73 ± 70	−37 ± 23	5 ± 2	−39 ± 23	67 ± 70
		**90**	−52 ± 19	5 ± 1	−77 ± 48	107 ± 54	−42 ± 22	5 ± 2	−75 ± 51	105 ± 57
		**98**	−25 ± 6	5 ± 1	−37 ± 15	35 ± 21	−24 ± 6	4 ± 1	−36 ± 16	34 ± 21
		**101**	−91 ± 32	9 ± 3	−81 ± 35	106 ± 59	−53 ± 47	6 ± 3	−54 ± 47	84 ± 76
ILE	***c5.300***	**6**	−39 ± 12	66 ± 15	--	--	−31 ± 19	24 ± 13	--	--
		**13**	−41 ± 21	53 ± 15	--	--	−30 ± 23	20 ± 15	--	--
		**17**	−56 ± 24	57 ± 18	--	--	−34 ± 16	35 ± 17	--	--
		**22**	−47 ± 16	61 ± 12	--	--	−46 ± 20	38 ± 14	--	--
		**23**	−50 ± 20	53 ± 18	--	--	−34 ± 11	41 ± 21	--	--
		**26**	−37 ± 14	51 ± 15	--	--	−24 ± 5	32 ± 13	--	--
		**67**	−25 ± 6	71 ± 12	--	--	−32 ± 8	39 ± 18	--	--
		**125**	−42 ± 24	60 ± 12	--	--	−33 ± 15	29 ± 12	--	--
		**141**	−38 ± 19	44 ± 14	--	--	−43 ± 17	16 ± 7	--	--
PHE	***c5.300***	**110**	−6 ± 3	17 ± 7	−3 ± 3	9 ± 3	−4 ± 2	9 ± 4	−2 ± 4	4 ± 4
		**145**	−5 ± 1	21 ± 5	−0 ± 1	3 ± 1	−5 ± 1	15 ± 7	−0 ± 1	3 ± 2
		**167**	−4 ± 1	13 ± 6	−0 ± 1	3 ± 1	−4 ± 1	8 ± 4	−0 ± 0	2 ± 1
		**172**	−4 ± 1	17 ± 4	−0 ± 0	3 ± 1	−4 ± 1	13 ± 4	0 ± 0	2 ± 1
		**202**	−3 ± 1	19 ± 8	0 ± 0	1 ± 1	−3 ± 1	9 ± 4	0 ± 0	1 ± 1
		**219**	−6 ± 1	−4 ± 20	−6 ± 7	11 ± 7	−4 ± 2	7 ± 3	−1 ± 2	2 ± 1
		**264**	−7 ± 3	19 ± 6	−2 ± 3	7 ± 2	−5 ± 3	11 ± 6	−1 ± 3	4 ± 3
		**286**	−5 ± 1	16 ± 5	−1 ± 1	5 ± 2	−4 ± 1	12 ± 6	−0 ± 1	4 ± 2
		**355**	−3 ± 1	14 ± 10	0 ± 0	1 ± 1	−3 ± 1	7 ± 5	0 ± 0	1 ± 1
		**634**	−5 ± 1	18 ± 4	0 ± 0	1 ± 1	−5 ± 1	13 ± 5	0 ± 0	2 ± 1
		**715**	−4 ± 1	20 ± 5	0 ± 0	3 ± 1	−4 ± 1	16 ± 6	0 ± 0	2 ± 1
		**753**	−5 ± 1	20 ± 5	0 ± 0	3 ± 1	−5 ± 1	14 ± 6	0 ± 0	2 ± 1
THR	***c5.300***	**6**	−64 ± 24	23 ± 5	−8 ± 8	45 ± 34	−49 ± 29	16 ± 7	−8 ± 8	37 ± 35
		**33**	−85 ± 24	38 ± 12	−8 ± 7	72 ± 36	−47 ± 34	21 ± 11	−8 ± 7	26 ± 19
		**52**	−57 ± 16	47 ± 11	−5 ± 3	29 ± 14	−39 ± 17	30 ± 14	−5 ± 3	26 ± 13
		**55**	−56 ± 22	42 ± 14	−10 ± 9	44 ± 32	−48 ± 21	31 ± 12	−10 ± 9	36 ± 17
		**60**	−20 ± 2	5 ± 1	−3 ± 2	20 ± 19	−18 ± 4	3 ± 2	−2 ± 2	19 ± 19
		**71**	−31 ± 12	17 ± 9	−8 ± 8	15 ± 10	−22 ± 13	8 ± 3	−8 ± 8	12 ± 10

^a^ Interaction character of a cluster is calculated as described in the text: Hyd(−) is unfavorable hydrophobic, Hyd(+) is favorable hydrophobic, Pol(−) is unfavorable polar, and Pol(+) is favorable polar; ^b^ ***Chess square.parse*** notation describes the residue’s backbone angle and χ_1_ bin (see text); ^c^ **Clusters** are named for the exemplar residue, i.e., closest to cluster centroid.

## Data Availability

As described in the text, all data used in this work were extracted from the Protein Data Bank of published X-ray crystal structures. Supporting Information Tables S1–S5 list the residue-level results for all calculations and all datasets described in this paper. All algorithms and formulas for the calculations we performed and reported are part of the HINT modeling system and are presented within this manuscript and/or its references. Requests for access to specific software code or extended data, some of which may require a non-disclosure agreement, should be made to the corresponding author.

## Supplementary Materials

The following supporting information can be downloaded at: https://www.mdpi.com/article/10.3390/molecules29122838/s1, Table S1: Soluble Cluster Data; Table S2: Membrane Soluble Domain Cluster Data; Table S3: Membrane Core Facing Cluster Data; Table S4: Membrane Lipid Facing ON Cluster Data; and Table S5: Membrane Lipid Facing OFF Cluster Data.
